# Different *csrA* Expression Levels in C versus K-12 *E*. *coli* Strains Affect Biofilm Formation and Impact the Regulatory Mechanism Presided by the CsrB and CsrC Small RNAs

**DOI:** 10.3390/microorganisms9051010

**Published:** 2021-05-07

**Authors:** Thomas Carzaniga, Federica A. Falchi, Francesca Forti, Davide Antoniani, Paolo Landini, Federica Briani

**Affiliations:** 1 Dipartimento di Bioscienze, Università degli Studi di Milano, 20133 Milan, Italy; thomas.carzaniga@unimi.it (T.C.); federica.falchi@unimi.it (F.A.F.); francesca.forti@unimi.it (F.F.); antoniani.d@gmail.com (D.A.); paolo.landini@unimi.it (P.L.); 2 Dipartimento di Biotecnologie Mediche e Medicina Traslazionale, Università degli Studi di Milano, Segrate, 20054 Milan, Italy

**Keywords:** CsrA, PNPase, *pgaABCD* operon, auto-aggregation, biofilm, poly-β-1,6-*N*-acetylglucosamine, sRNA-dependent regulation

## Abstract

*Escherichia coli* C is a strong biofilm producer in comparison to *E. coli* K-12 laboratory strains due to higher expression of the *pgaABCD* operon encoding the enzymes for the biosynthesis of the extracellular polysaccharide poly-β-1,6-*N*-acetylglucosamine (PNAG). The *pgaABCD* operon is negatively regulated at the post-transcriptional level by two factors, namely CsrA, a conserved RNA-binding protein controlling multiple pathways, and the RNA exonuclease polynucleotide phosphorylase (PNPase). In this work, we investigated the molecular bases of different PNAG production in C-1a and MG1655 strains taken as representative of *E. coli* C and K-12 strains, respectively. We found that *pgaABCD* operon expression is significantly lower in MG1655 than in C-1a; consistently, CsrA protein levels were much higher in MG1655. In contrast, we show that the negative effect exerted by PNPase on *pgaABCD* expression is much stronger in C-1a than in MG1655. The amount of CsrA and of the small RNAs CsrB, CsrC, and McaS sRNAs regulating CsrA activity is dramatically different in the two strains, whereas PNPase level is similar. Finally, the compensatory regulation acting between CsrB and CsrC in MG1655 does not occur in *E. coli* C. Our results suggest that PNPase preserves CsrA-dependent regulation by indirectly modulating *csrA* expression.

## 1. Introduction

The Csr/Rsm (carbon storage regulator/repressor of stationary phase metabolites) system controls key phenotypes ranging from carbon metabolism to virulence and biofilm formation in Gammaproteobacteria. Over the years, studies by different groups, and in particular by T. Romeo’s lab, have clarified many aspects of the mechanism by which the protein CsrA (or its ortholog RsmA) controls this system in *Escherichia coli* and other bacteria, showing that CsrA and its orthologs act by modulating translation, decay and transcription elongation of a number of mRNAs [1,2]. CsrA homodimers bind GGA motifs located in a single-stranded loop of short hairpins usually present in multiple copies in the 5′ untranslated regions (5′-UTR) of CsrA mRNA targets [3,4]. CsrA protein activity is negatively regulated by the non-coding small RNAs (sRNAs) CsrB and CsrC, which contain multiple CsrA binding sites (i.e., 18 in CsrB and 14 in CsrC) and antagonize CsrA activity by sequestering it [5,6]. Another sRNA, namely McaS, also binds CsrA and negatively regulates its activity [7]. Multiple positive and negative feedback loops strictly control the amounts of CsrA and its sRNA regulators [8,9]. CsrA indirectly activates *csrB* and *csrC* transcription [10,11] and stabilizes CsrB and CsrC by repressing the expression of *csrD*, which promotes CsrB and CsrC RNase E-dependent degradation [12]. Coordinated regulation also exists between the expression level of CsrB and CsrC, as the absence of each of them elicits compensatory effects on the expression of the other one [6,12].

**Figure 1:**
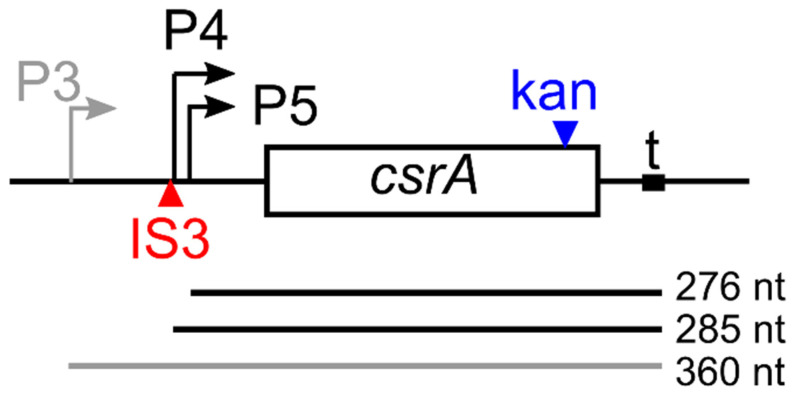
*csrA* locus and *csrA* mRNAs. The 186 bp long open reading frame (ORF; box) of the *csrA* gene and the transcription start sites for P3, P4, and P5 promoters (bent arrows) located 137, 62, and 53 bp upstream of the ATG start codon, respectively, are represented [13]. Red and blue arrowheads, insertion points of IS3 in C-1a and of the kan^R^ cassette in the *csrA*::kan mutants [14,15]. The region between positions +8 and +37 downstream of the *csrA* stop codon (t) was identified as a putative termination site [16]. mRNAs originating from P3 (gray line), P4, and P5 (black lines) and terminating at +37 with respect to the *csrA* stop codon are reported with their predicted length.

In *E. coli* K-12, the *csrA* gene can be transcribed by multiple promoters (Figure 1), among which the Eσ^D^- dependent P5 and the Eσ^S^- dependent P3 are the most active in exponential and stationary phase, respectively. CsrA indirectly activates transcription at P3 and negatively regulates the translation of the P3 mRNA. Conversely, the transcript starting at P5 does not seem to be subject to CsrA translation modulation [13]. The *csrA* promoter region is altered in *E. coli* C strains because of the insertion of the IS3 transposable element within the −35 region of the P4 promoter (Figure 1). This insertion abolishes *csrA* autoregulation acting on the P3 transcript. Indeed, in *E. coli* C, transcripts starting from all *csrA* promoters but P5, which is located immediately downstream of the IS3 insertion site, are predicted to terminate within IS3, and thus *csrA* is transcribed exclusively from P5 [14].

The *pgaABCD* operon is one of the targets of CsrA in *E. coli* [17,18]. *pgaABCD* encodes the enzymes for the biosynthesis of poly-β-1,6-*N*-acetylglucosamine (PNAG), an exopolysaccharide with a major role as an extracellular matrix component in biofilms of both Gram-positive and Gram-negative bacteria [19,20,21]. The *pgaABCD* operon expression is positively regulated by the transcription activator NhaR and negatively regulated by CsrA, which binds multiple sites in its 5′-untranslated region (5′-UTR) and causes premature transcription termination and *pgaA* translation repression [18,22,23,24]. Surprisingly, we observed that *pgaABCD* operon expression and PNAG production were increased in C-1a Δ*csrB* and Δ*csrC* mutants [25]. Since free CsrA (i.e., not sequestered by CsrB/CsrC) should be enhanced in these mutants, this observation is difficult to reconcile with the current model of *pgaABCD* negative regulation by CsrA [17].

**Figure 2:**
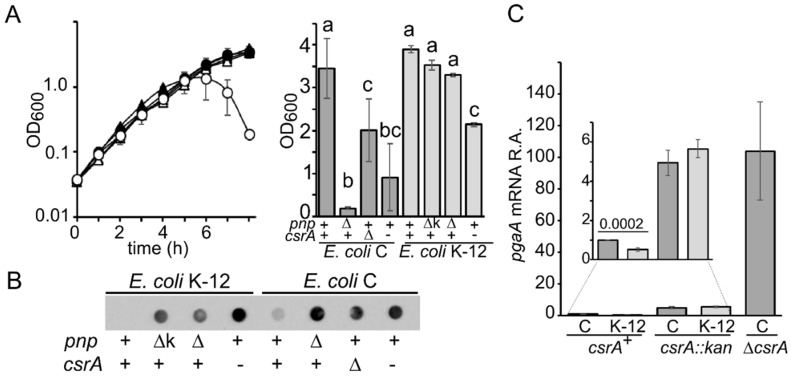
Growth, PNAG production, and *pgaA* expression in *E. coli* C and K-12 strains with *pnp* and *csrA* mutations. (**A**), left panel. Growth at 37 °C in M9-LG monitored by measuring the OD_600_ of cultures of the following strains: C-1a (black circles); C-5691 (Δ*pnp*; empty circles); MG1655 (black triangles); KG-206 (Δ*pnp*::kan; gray triangles); KG-211 (Δ*pnp;* empty triangles). (**A**) Right panel. OD_600_ of cultures incubated 8 h at 37 °C in M9-LG. All cultures at time = 0 had OD_600_ of about 0.04. *E. coli* C strains: *csrA*^+^ *pnp*^+^, C-1a; *csrA*^+^ Δ*pnp*, C-5691; Δ*csrA pnp*^+^, C-5939; *csrA^-^ pnp*^+^, C-5741. *E. coli* K-12 strains: *csrA*^+^ *pnp*^+^, MG1655; *csrA*^+^ Δ*pnp:kan* (Δk), KG-206; *csrA*^+^ Δ*pnp,* KG-211; *csrA^-^ pnp*^+^, MG1655 *csrA*::kan. Bars represent means (*n* = 3) with standard deviation. Letters above the bars refer to the results of ANOVA with the Tuckey post-hoc test (*p* < 0.0001). Means sharing a letter are not statistically different. (**B**) PNAG was extracted from 1.5 OD_600_ of overnight cultures of the strains listed in the description of panel (**A**), right panel. 1/30 of cell lysate (10 µL) was spotted onto a nitrocellulose membrane and immunodecorated with PNAG-specific antibodies. (**C**) *pgaA* mRNA relative expression (R.A.) with respect to that in C-1a (C *csrA*^+^). qRT-PCR of RNA extracted from cultures grown at 37 °C in M9-LG up to OD_600_ = 0.5 was performed as described in Materials and Methods. Bars represent the average with range of determinations on 3 (*csrA*^+^ and Δ*csrA* strains) or 2 (*csrA*::kan strains) cultures. The significance of the difference between the average expression in C-1a and MG1655 (K-12 *csrA*^+^) is reported in the inset and was estimated with a *t*-test.

Besides CsrA, another negative regulator of *pgaABCD* expression is the RNA exonuclease polynucleotide phosphorylase (PNPase) [26], which also acts at the post-transcriptional level. Mechanism of PNPase-dependent *pgaABCD* regulation is unclear, but in cis determinants of PNPase-dependent regulation, as well as for CsrA, lie in the *pgaABCD* 5′-UTR [25]. Consistent with the role of PNPase as a negative regulator of *pgaABCD*, the deletion of the *pnp* gene encoding PNPase in the *E. coli* K-12 strain MG1655 determines increased adhesion, a phenotype suppressed by the deletion of the *pgaA* gene [25]. The effect of the Δ*pnp* mutation is stronger in the *E. coli C* C-1a genetic background, as cultures in the minimal medium of a C-1a Δ*pnp* mutant undergo massive aggregation due to *pgaABCD* operon overexpression and PNAG hyperproduction, whereas MG1655 Δ*pnp* cultures do not visibly aggregate in the same conditions (see Figure 2A in the Results section) [25], suggesting that they may produce less PNAG. In agreement with this hypothesis, it was recently shown that *E. coli* C produces more robust biofilm than other *E. coli* strains, among which *E. coli* K-12 [14].

In this work, we investigated the molecular bases of different PNAG production in C-1a and MG1655 as models of *E. coli* C and K-12 strains, respectively. We found that the *pgaABCD* operon is more tightly regulated by CsrA in MG1655 than in C-1a. Conversely, the negative effect exerted by PNPase on *pgaABCD* expression is much stronger in *E. coli* C than in *E. coli* K-12. The sRNAs regulating CsrA activity have different expression profiles in the two strains. We discuss the hypothesis that both different *pgaABCD* expression in C-1a *vs*. MG1655, as well as negative CsrB and CsrC effects on *pgaABCD* observed in *E. coli* C, may be the consequence of adaptation to different CsrA levels found in the two genetic backgrounds.

## 2. Materials and Methods

### 2.1. Bacteria, Plasmids, and Growth Media

Bacterial strains and plasmids are listed in Table 1. Strains were constructed either by λ Red-mediated recombination or P1 transduction [27,28]. Oligonucleotides FG2624-FG2625 (amplicon Δ*csrA*::kan), FG2524-FG2525 (amplicon Δ*csrB*::kan), and FG2585-FG2586 (amplicon Δ*csrC*::cat) (Table S1) were used to PCR- amplify DNA fragments with terminal ends homologous to the bacterial chromosome and containing the kanamycin (kan) resistance cassette of pKD13 or the chloramphenicol (cat) resistance cassette of pKD3. The antibiotic cassettes were removed by FLP-FRT mediated recombination [27]. pCSRA was constructed by cloning in pGZ119HE digested with *Hind*III and *Eco*RI the *csrA* gene (from −23 to +32 with respect to the open reading frame (ORF) start and stop codon, respectively) obtained by PCR amplification with primers FG3151-FG3152 on MG1655 genome. Bacterial cultures were grown at 37 °C in LD broth (1% tryptone, 0.5% yeast extract and 0.5% NaCl) or M9-LG medium (82 mM Na_2_HPO_4,_ 24 mM KH_2_PO_4_, 85 mM NaCl, 19 mM NH_4_Cl, 1 mM MgSO_4_, 0.1 mM CaCl_2_, supplemented with 0.25 g/L tryptone, 0.125 g/L yeast extract, 0.125 g/L NaCl, 0.4% glucose) supplemented with antibiotics when needed and grown to OD_600_ = 0.8, if not otherwise indicated.

### 2.2. Gene Expression Determination

Basic procedures for RNA extraction, Northern blot analysis, and synthesis of radiolabeled riboprobes by in vitro transcription with T7 RNA polymerase were previously described [36,37]. The DNA template for *csrA* riboprobe synthesis was amplified by PCR on genomic DNA with oligonucleotides FG2647 and PL191. The oligonucleotides for CsrB (FG2530, csrB-5′; FG2531, csrB-3′; PL208, csrB-m), CsrC (FG2568) and McaS (FG2753) were 5′-end-labeled with T4 polynucleotide kinase in the presence of [γ-^32^P]ATP [38]. The conditions of hybridization with oligonucleotides were described previously [39]. Autoradiographic images and densitometric analysis of Northern blots were obtained by phosphorimaging using ImageQuant software (Molecular Dynamics). Quantitative (real-time) reverse transcriptase PCR (qRT-PCR) was performed as described [40]. Oligonucleotides PL101 and PL102 were used for 16S rRNA reverse transcription and PCR amplification.

### 2.3. Luciferase Activity Assay

Bacterial cultures were grown at 37 °C in LD broth supplemented with ampicillin 50 µg/mL up to OD_600_ = 0.5. A total of 5 mL of cultures were harvested by centrifugation 5 min at 4000× rpm and 4 °C, and the bacterial pellet was resuspended in 5 mL of M9-LG broth supplemented with ampicillin 50 µg/mL. Bacteria were grown 90 min at 37 °C, harvested by centrifugation 5 min at 4000× rpm and 4 °C and resuspended in PBS at OD_600_ = 0.1. To measure luciferase activity, 5 µL of bacterial suspension were diluted in 500 µL of fresh PBS, and 20 µL of 1% decanal in ethanol was added. Luminescence was measured with a Stratec luminometer.

### 2.4. PNAG Detection

PNAG production was determined as described [25]. Bacteria were grown overnight in M9-LG at 37 °C. A total of 1.5 OD_600_ were collected, and 1/30 of cell lysate (10 μL) was spotted onto a nitrocellulose filter using a Dot-blot apparatus (Bio-Rad, Hercules, CA, USA), incubated overnight at 4 °C with PNAG antibodies (a kind gift from G.B. Pier [41]) and revealed using ECL Western blotting reagent PDS Standard (Genespin, Milano, Italy).

### 2.5. Western Blotting

*E. coli* crude extracts were obtained as described previously [42]. Protein content was determined using Coomassie Plus protein assay reagent (Pierce, Thermo Scientific, Waltham, MA, USA). Sodium dodecyl sulfate polyacrylamide gel electrophoresis (SDS-PAGE) was performed on 10% resolving gels containing 0.1% SDS. PageRuler Prestained Molecular-weight markers (Thermo Scientific, Waltham, MA, USA) were used as a size reference. For immunological detection of PNPase and CsrA, the gels were blotted onto a nitrocellulose (Hybond ECL, SIGMA, Saint Louis, MO, USA) sheet and incubated with polyclonal anti-PNPase [43] and anti-*csrA* antibodies (Biorbyt Ltd., Cambridge, UK), respectively. Since the anti-*csrA* antibody provided many strong unspecific signals, we preincubated a 1:500 antibody dilution with 0.15 mg/mL of Δ*csrA* extract for 2 h at 4 °C before using it for filter immunodecoration. Immunoreactive bands were revealed using the ECL Western blotting reagent PDS Standard (Genespin, Milano, Italy).

### 2.6. Statistical Analysis

Statistical tests were applied to compare the means of results obtained by analyzing at least three biological replicates for each group/condition. We used one-way analysis of variance (ANOVA) with Tukey’s post-hoc test for comparison among means of three or more groups and an independent two-tailed *t*-test for comparison between two groups.

## 3. Results

### 3.1. E. coli C Produces More PNAG than E. coli K-12

The *pnp* gene deletion causes massive aggregation in *E. coli C* growing in M9-LG medium [25], whereas in *E. coli* K-12 has no apparent effect (Figure 2A). This difference can be explained by lower PNAG production in *E. coli* K-12 *pnp*^+^ and Δ*pnp* strains with respect to their *E. coli* C counterparts [14] (Figure 2B). In both genetic backgrounds, *csrA* mutations such as the *csrA* gene deletion [25] or a *csrA* hypomorphic allele encoding a partially active protein (i.e., *csrA*::kan allele [15,44]) enhanced PNAG production, as expected, and slowed the growth after about 3–4 generations in C-1a and 5–6 in MG1655 (Figure 2A; Supplementary Figure S1).

It should be mentioned that around the 20% of cultures obtained by inoculating C-1a Δ*csrA* colonies in LD broth showed no/poor growth, whereas C-1a *csrA*::kan cultures did not show grow defects in LD but had erratic growth rate upon dilution in M9-LG (Supplementary Figure S1), suggesting genetic variability. Inconsistency in growth rate upon dilution in M9-LG was not observed with MG1655 *csrA*::kan (Figure 2A) [44].

### 3.2. CsrA-Dependent Regulation of pgaABCD Operon Is More Stringent in E. coli K-12 than in E. coli C

Increased PNAG production in C-1a with respect to MG1655 may be due to enhanced expression of *pgaABCD* biosynthetic operon in the former strain. Consistent with this hypothesis, the *pgaA* mRNA was about two-fold more abundant in C-1a than in MG1655 (Figure 2C). This did not depend on the higher activity of the *pgaABCD* promoter in C-1a, as transcription efficiency from the *pgaABCD* promoter was comparable in the two strains (Supplementary Figure S2).

Since *pgaABCD* mRNA level is subject to post-transcriptional control by CsrA, we checked whether CsrA was responsible for the *pgaABCD* expression differential between C-1a and MG1655. We found that the amount of *pgaA* mRNA (taken as representative of the operon mRNA) in the *csrA*::kan mutants of the two strains was similar and enhanced with respect to the *csrA*^+^ strains. *pgaA* mRNA level was further increased in the C-1a Δ*csrA* mutant (Figure 2C). Thus, in both genetic backgrounds, CsrA negatively regulates *pgaA* expression, and it appears to be responsible for the difference in *pgaA* expression between *E. coli* C and K-12. To strengthen this hypothesis, since CsrA down-regulates *pgaA* translation by interacting with its 5′-UTR, we assayed the expression of a *pgaA-lux* translational fusion between the promoter region and 5′-UTR of *pgaABCD* and the luciferase gene in C-1a and MG1655 and in their respective Δ*csrA* derivatives. All strains had the Δ*pgaC* mutation in the chromosome to avoid auto-aggregation due to PNAG over-production and Δ*csrA* suppressor selection [7,25,45]. We found that luciferase activity was ten-fold higher in C-1a than in MG1655 (Table 2). The Δ*csrA* mutation resulted in a ca. 26-fold relative induction in C-1a and in a staggering 420-fold relative induction in K-12, boosting luciferase activity to comparable levels in *E. coli* C and K-12 strains, and thus strongly supporting the hypothesis that *pgaABCD* expression is lower in MG1655 than in C-1a because CsrA-dependent repression is tighter in the former strain.

### 3.3. PNPase-Dependent Regulation of pga Operon Is More Stringent in E. coli C than in E. coli K-12

We compared the contribution of PNPase to *pgaABCD* regulation in *E. coli* C and K-12 by exploiting the *pgaA*-lux fusion described above. Luciferase activity was enhanced around 14-fold- in C-1a Δ*pnp*, in agreement with previously published data [25], and 3-fold in MG1655 Δ*pnp* with respect to their *pnp*^+^ counterparts. Thus, in the presence of CsrA, PNPase negative effect on *pgaABCD* expression is stronger in *E. coli* C than in K-12. The additional Δ*pnp* mutation in the Δ*csrA* strains enhanced luciferase activity in *E. coli* C. In *E. coli* K-12, an increment was also observed, but this result is less convincing because of the high variability associated with MG1655 Δ*csrA* derivatives in this assay (Table 2).

**Figure 3:**
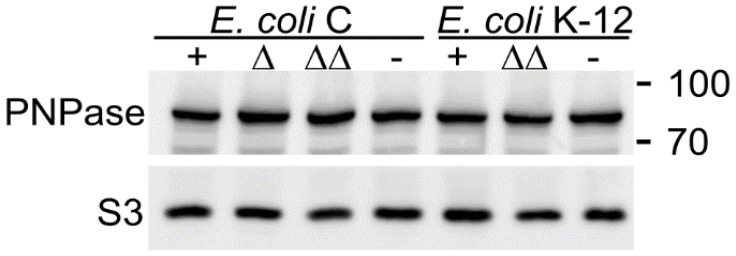
PNPase levels in *E. coli* C and K-12 *csrA* mutants. Western blotting of proteins extracted from cultures grown up to OD_600_ = 0.8 in M9-LG at 37 °C of *E. coli* C strains C-1a (*+*), C-5939 (Δ; Δ*csrA*), C-5738 (ΔΔ; Δ*csrA* Δ*pgaC*); C-5741 (−; *csrA^−^*) and *E. coli* K-12 MG1655 (+; *csrA*^+^), KG-294 (ΔΔ; Δ*csrA* Δ*pgaC*), MG1655 *csrA*::kan (−; *csrA^−^*). Proteins (15 µg) were run on a 10% polyacrylamide-SDS gel, blotted onto a nitrocellulose membrane, and hybridized with anti-PNPase and anti-S3 antibodies. The position and MW (in KDa) of bands of the PageRuler Prestained Protein Ladder (Thermo Scientific) are reported on the right.

It was reported that CsrA regulates PNPase translation by binding to *pnp* mRNA 5′-UTR [46]. However, the PNPase level was the same between *E. coli* C and K-12 and also between *csrA*^+^ and *csrA* mutants (Figure 3). Thus, the higher impact of PNPase on *pgaABCD* regulation in *E. coli* C vs. K-12 does not depend on differences in *pnp* expression.

### 3.4. Expression Profile of the csrA Gene and of sRNAs Regulating CsrA Activity in E. coli C and K-12

**Figure 4:**
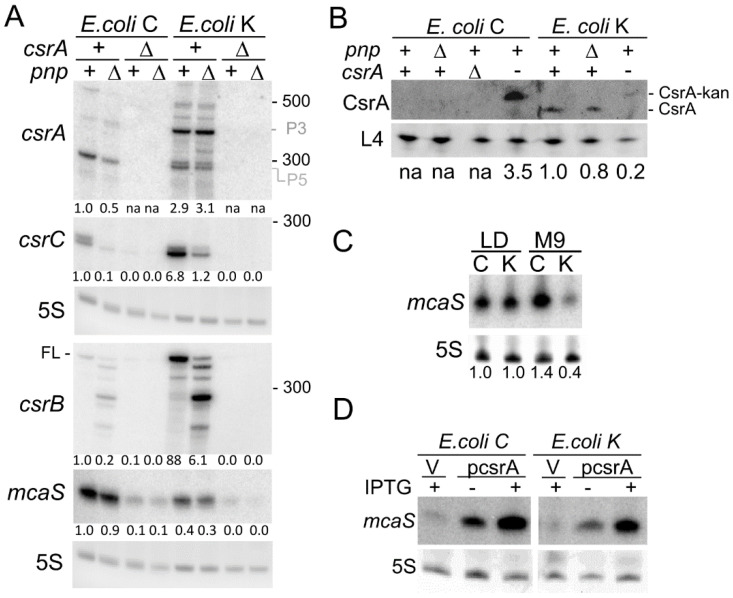
Expression of genes encoding *pgaABCD* regulators in *E. coli* C and K-12. (**A**,**C**,**D**). Northern blotting of RNA extracted from cultures grown to OD_600_ = 0.8 at 37 °C. RNA samples (10 µg) were loaded on 6% polyacrylamide-urea gel, blotted onto a nylon membrane, and hybridized with the CSRA riboprobe (**A**, upper panel) or radiolabeled oligonucleotides specific for the CsrC, CsrB, and McaS sRNAs (**A**,**C**,**D**, indicated on the left of the panels with the respective gene name). 5S, 5S rRNA used as a gel loading control. Figures under the panels refer to signal quantification with ImageQuant. The signals were normalized for 5S signals and for the signals obtained in C-5936 (**A**) or C-1a LD cultures (**C**). (**A**) RNA extracted from cultures in M9-LG of *E. coli* C C-5936 (*csrA^+^pnp^+^*), C-5937 (*csrA^+^*Δ*pnp*), C-5738 (Δ*csrA pnp^+^*) and C-5739 (Δ*csrA* Δ*pnp*); *E. coli* K-12 KG-292 (*csrA^+^pnp^+^*), KG-293 (*csrA^+^*Δ*pnp*), KG-294 (Δ*csrA pnp^+^*) and KG-295 (Δ*csrA* Δ*pnp*). All strains contained the Δ*pgaC* mutation. The position and MW (in nt) of bands of the low-range ssRNA ladder (NEB) are reported on the right. The estimated size of McaS based on the migration of MW markers was ca. 96 nt as expected [47]. P3 and P5, transcripts putatively starting at P3 and P5 promoters; FL, full-length CsrB. (**C**) RNA extracted from cultures of C-1a (**C**) and MG1655 (K) grown in LD or M9- LG (M9) as indicated. (**D**) RNA extracted from cultures of C-1a (*E. coli* C) and MG1655 (*E. coli* K-12) carrying plasmid pCSRA, as indicated, or the empty vector pGZ119 (V). 1 mM IPTG was present in the M9-LG medium if indicated (+). (**B**) Western blotting of proteins extracted from cultures grown up to OD_600_ = 0.8 in M9-LG at 37 °C of *E. coli* C strains C-1a (*csrA^+^pnp^+^*), C-5691 (*csrA^+^*Δ*pnp*), C-5939 (Δ*csrA pnp^+^*), C-5741 (*csrA^-^ pnp^+^*); and *E. coli* K-12 MG1655 (*csrA^+^pnp^+^*), KG-211 (*csrA^+^*Δ*pnp*), MG1655 *csrA*::kan (*csrA^-^ pnp^+^*). Proteins (15 µg) were run on a 16% tricine gel, blotted onto a nitrocellulose membrane, and hybridized with the anti-CsrA antibody and the anti-L4 antibody as the loading control. The results of CsrA signal quantification with ImageQuant normalized for L4 signals are shown below the lanes. The value obtained in MG1655 was taken as a reference for comparison. na, not applicable.

We analyzed *csrA* gene expression *in E. coli* C and K-12 by Northern blotting. In MG1655, the main *csrA* signal corresponded to an RNA with an estimated length of 350–370 nt, compatible with an mRNA originating from the P3 promoter and terminating 30–50 nt downstream of the *csrA* stop codon (Figure 1 and Figure 4A). Two mRNAs of similar length and migrating slightly faster than the 300 nt long RNA marker were also present. These species likely correspond to transcripts starting at P4/P5 and terminating where P3 mRNA also ends. In C-1a, the main signal corresponded to the putative P5 mRNA, together with faint bands corresponding to longer RNAs. The abundance of *csrA* mRNAs, considering the overall amount of P3, P4, and P5 mRNAs for MG1655 and P5 mRNA for C-1a, was around three-fold higher in *E. coli* K-12 than in *E. coli* C.

**Figure 5:**
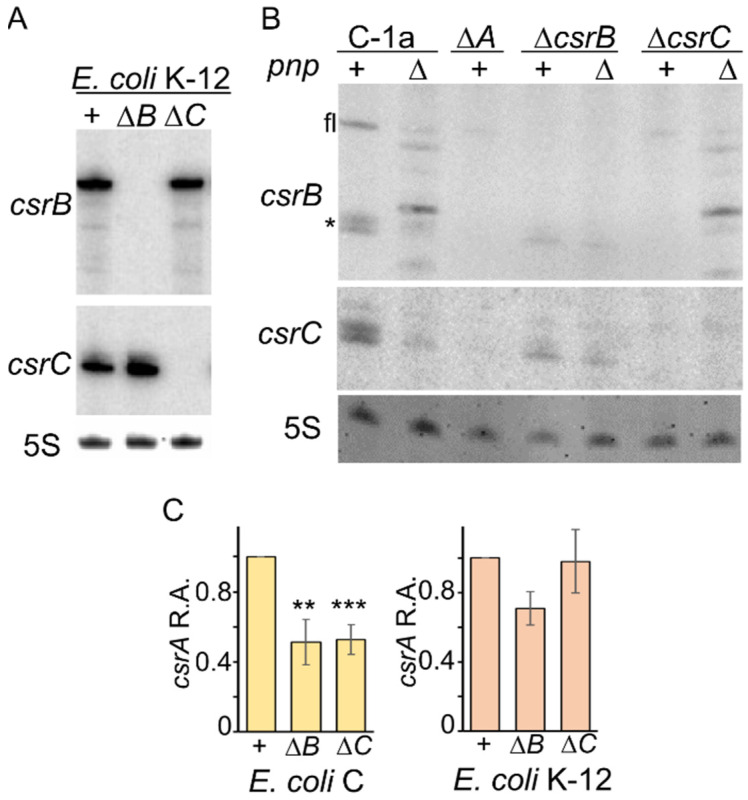
Expression of *csrA*, *csrB* and *csrC* in Δ*csrB* and Δ*csrC* mutants. Northern blotting of RNA extracted from cultures grown up to OD_600_ = 0.8 in M9-LG at 37 °C. RNA samples (10 µg) were loaded on 6% polyacrylamide-urea gel, blotted onto a nylon membrane, and hybridized with radiolabeled oligonucleotides specific for the CsrB (PL208), and CsrC (FG2568) sRNAs (indicated on the left of the panels with the respective gene name). The CsrB oligonucleotides cross-hybridizes with CsrC (*). 5S, 5S rRNA used as gel loading control. FL, full-length CsrB. (**A**) RNA extracted from strains MG1655 (+), KG-303 (Δ*csrB*; Δ*B*), KG-300 (Δ*csrC*; Δ*C*). (**B**) RNA extracted from strains C-1a (C-1a, +), C-5691 (C-1a, Δ), C-5938 (Δ*csrA*; Δ*A*) C-5941 (Δ*csrB*, +), C-5943 (Δ*csrB*, Δ), C-5740 (Δ*csrC*, +), C-5946 (Δ*csrC*, Δ). (**C**), left panel. RNA extracted from triplicate cultures of C-1a (+), C-5941 (Δ*csrB*; Δ*B*), C-5740 (Δ*csrC*; Δ*C*). Bars represent average with standard deviation of quantification with ImageQuant of the P5 mRNA signal observed in Northern blot experiments. Stars refer to *t*-test results (**, *p* < 0.01; *** *p* < 0.001). C, right panel. RNA extracted from duplicate cultures of MG-1655 (+), KG-303 (Δ*csrB*; Δ*B*), KG-300 (Δ*csrC*; Δ*C*). Bars represent average with range of the sum of P3 and P4/P5 *csrA* mRNA signals.

These results were confirmed also in C-1a and MG1655 strains not containing the Δ*pgaC* mutation (Table 3; see also Figure 5A). No signal was detected in the Δ*csrA* strains, as expected.

The Δ*pnp* mutation in the C-1a background decreased *csrA* mRNA abundance, whereas it did not significantly change the overall abundance of *csrA* transcripts when present in MG1655 (Figure 4A).

Consistent with the *csrA* transcription profile, western blotting analysis showed that the CsrA level was higher in MG1655 than in C-1a (Figure 4B). Indeed, we could not detect CsrA in any tested *E. coli* C strains, with the paradoxical exception of the *csrA*::kan mutant, in which the signal corresponding to a possible chimeric protein slightly bigger than wildtype CsrA [10] was stronger than in MG1655 *csrA*::kan (Figure 4B). C-1a *csrA*::kan was obtained by P1-mediated transduction from MG1655 *csrA*::kan, implying that the *csrA* promoter region in C-1a *csrA*::kan is in all probability deriving from the donor MG1655. Consistent with *csrA* locus transcription from the same promoter in the two mutant strains, *csrA* mRNAs with the same electrophoretic mobility, and thus presumably of the same length, are produced in C-1a and MG1655 *csrA*::kan mutants (Supplementary Figure S3A). Given the growth variability shown by C-1a *csrA*::kan mutants in M9-LG (Figure 2A and Supplementary Figure S1), it is possible that in (some) C-1a *csrA*::kan cultures, suppressor mutations may result in an increased amount of the CsrA-kan chimeric protein.

The amount of sRNAs CsrB, CsrC, and McaS were dramatically different between C-1a and MG1655. CsrC and especially CsrB were much more abundant, and McaS strongly reduced in MG1655 with respect to C-1a (Figure 4A; Table 3).

McaS transcription was previously reported to be activated in low glucose [47,48]. We thus compared its levels in bacteria growing in either LD broth, in which glucose is scarce and quickly consumed by growing bacteria [49], or M9-LG, which contains 0.4% glucose. As expected, in MG1655, McaS was more abundant in the LD medium than in M9-LG. Conversely, the McaS amount was comparable in C-1a cultures growing in either media (Figure 4C).

The Δ*pnp* mutation had similar effects on CsrB, CsrC, and McaS in *E. coli* C and K-12 strains. It decreased CsrC and, to a lesser extent, McaS sRNAs abundance. Concerning CsrB, a nearly identical RNA pattern was found in *E. coli* C and K-12 Δ*pnp* strains, with a strong reduction in the full-length RNA and accumulation of shorter species (Figure 4A and Table 3). Hybridization with oligonucleotides complementary to either the CsrB 5′- or the 3′-end (Supplementary Figure S3B) confirmed that these RNAs are CsrB degradation products mainly shortened at the 3′-end as already found in *E. coli* K-12 and *Salmonella* [50,51].

In Δ*csrA* and Δ*csrA* Δ*pnp* mutants, all sRNAs, and in particular CsrB and CsrC, were less expressed. Indeed, faint CsrB and CsrC signals corresponding to full-length transcripts were visible only upon long exposition of the filters (Figure 4A and data not shown). These results were consistent with previous evidence showing that CsrA positively regulates *csrB* and *csrC* expression [6,10]. As for McaS, in contrast with our data, its level was reported to be similar in MG1655 and in its isogenic *csrA*::kan mutant [7]. To further check whether the McaS amount is modulated by CsrA, we analyzed the effect of ectopic *csrA* expression from a plasmid on McaS production in C-1a and MG1655. As shown in Figure 4D, McaS was more abundant in strains with plasmid pCSRA, which carries the *csrA* gene under the *ptac* promoter, than in those with the empty vector, and its amount further increased upon induction of *csrA* transcription with IPTG. Thus, CsrA positively controls the McaS level.

### 3.5. Compensatory Regulation of CsrB and CsrC Does Not Occur in E. coli C

According to literature data, the amount of CsrB and CsrC increases in MG1655 mutants with either *csrC* or *csrB* null mutations, respectively, a mechanism that compensates the lack of either sRNA by increasing the amount of the other one [6]. Consistent with these observations, we found around a 2.5-fold increase in CsrC in the MG1655 Δ*csrB* with respect to the *csrB*^+^. On the contrary, the CsrB amount was unchanged in the presence or absence of the *csrC* gene (Figure 5A), showing that compensatory regulation takes place only for the Δ*csrB* strain in our experimental conditions. To assess whether this regulation also occurs in *E. coli* C, we analyzed CsrB and CsrC levels in C-1a Δ*csrC* and Δ*csrB* mutants, respectively. Surprisingly, CsrB decreased to 0.2 ± 0.04 in the Δ*csrC* strain and CsrC to 0.01 ± 0.02 in the Δ*csrB* mutant compared with their levels in C-1a (Figure 5B). Thus, in *E. coli* C, the absence of CsrB or CsrC negatively affects the expression of the other one. In double Δ*csrB* Δ*pnp* or Δ*csrC* Δ*pnp* mutants, the expression of *csrC* and *csrB*, respectively, was similar to that found in the single Δ*pnp* mutant (Figure 5B).

The level of *csrA* mRNA was two-fold higher in C-1a than in *csrB* or *csrC* defective mutants (Figure 5C, left panel). As for *E. coli* K-12, the *csrA* mRNA, and in particular, the P3 transcript, was reduced by about 20% in the Δ*csrB* mutant, whereas no effect was observed in the Δ*csrC* (Figure 5C, right panel).

### 3.6. Ectopically Expressed RNase II Restores CsrB and CsrC Production in C-1a Δpnp

We previously found that ectopic expression of the *rnb* gene encoding RNase II from a plasmid suppressed auto-aggregation in C-1a Δ*pnp*. The suppression was specifically elicited by RNase II, as overexpression of *rnr* encoding the other *E. coli* exonuclease, namely RNase R, did not prevent aggregation [25]. We looked at CsrB and CsrC levels in strains overexpressing the two exonucleases to evaluate whether they have a different impact on the expression of these sRNAs.

**Figure 6:**
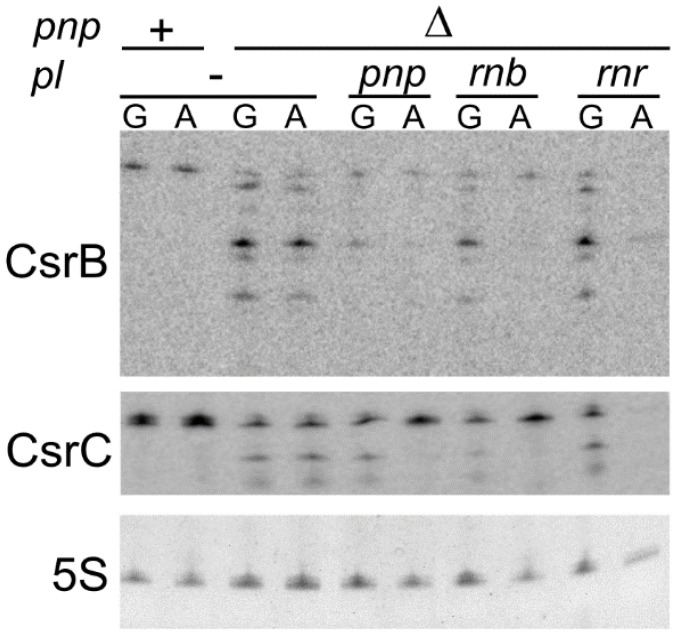
Expression of *rnb* restores CsrB and CsrC production in a Δ*pnp* strain. Northern blotting of RNA extracted from cultures grown up to OD_600_ = 0.8 in M9-LG at 37 °C. Bacteria were pelleted, washed, split in two flasks containing the same volume of M9 supplemented with 2.5%LD and either 0.4% glucose (G) or 1% arabinose (A) and incubated 45 min at 37 °C before RNA extraction. RNA samples (10 µg) were loaded on 6% polyacrylamide-urea gel, blotted onto a nylon membrane, and hybridized with radiolabeled oligonucleotides specific for the CsrB (PL208) and CsrC (FG2568) sRNAs (indicated on the left of the panels with the respective gene name). 5S, 5S rRNA used as the gel loading control.

As shown in Figure 6, we found that ectopic expression of all exonucleases caused the complete disappearance of signals corresponding to CsrB and CsrC degradation products, but only PNPase and RNase II partially restored CsrB and CsrC full-length production, whereas RNase R did not.

## 4. Discussion

In this work, we show that CsrA is expressed at a low level in *E. coli* C because of impaired transcription caused by the insertion of IS3 in the *csrA* P4 promoter [14]. Transcripts starting at upstream promoters end presumably within the transposon, and only P5 mRNA is produced. Transposons are major drivers in evolution [52,53]. In fact, by integrating at multiple positions within a genome, they may stimulate genome rearrangements through homologous recombination. Moreover, they can have a deep impact on gene expression by inserting within coding or regulatory regions, which in turn may deeply affect bacterial physiology. The insertion of the IS3 transposable element into the *csrA* promoter of *E. coli* C may be considered a textbook case in this respect, as by downregulating *csrA* expression, it determines increased *pgaABCD* expression that, consequently, stimulates auto-aggregation and biofilm formation.

Not only CsrA but also the molecular decoys modulating its activity, namely the sRNAs CsrB, CsrC, and McaS, are expressed at different levels in C-1a with respect to MG1655. In particular, CsrB and CsrC are more than fifty- and six-fold more abundant, respectively, in MG1655 than in C-1a, whereas McaS is less abundant. This is not due to differences in their genes as the sequences of the *csrB*, *csrC,* and *mcaS* loci, including the intergenic 200 pb regions upstream overlapping their promoters, are identical between C-1a and MG1655. The same also applies to *uvrY* and *barA* genes that are involved in CsrB and C regulation (data not shown). It seems likely that low CsrB and CsrC expression in C-1a may be a consequence of low CsrA levels. In fact, *csrA* deletion almost completely abolishes *csrB* and *csrC* expression in *E. coli* C (and K-12), suggesting that the indirect transcriptional activation of CsrB and CsrC by CsrA operating in *E. coli* K-12 [6,10] is maintained in *E. coli* C.

Concerning *mcaS*, we found that it is expressed at comparable levels in C-1a and MG1655 cultures growing in LD broth, which contains very little glucose [49]. In M9-LG, which contains a higher glucose concentration, *mcaS* expression drops in MG1655 while remaining high in C-1a. Thus, the expression profile of McaS is consistent with its reported regulation by glucose [47,48] in MG1655 but not in C-1a, further highlighting differences in sRNA expression between the two strains. We do not have an explanation for the high McaS level in C-1a in the presence of glucose, which may be due to transcription activation by factors different than CRP-cAMP, which controls catabolite repression in *E. coli* [54]. It should be mentioned that also in *E. coli* K-12, glucose-dependent *mcaS* regulation seems to be only partially dependent on CRP [48]. Post-transcriptional mechanisms modulating McaS stability can also play a role in determining its expression profiles in the two strains. For instance, the *csgD* mRNA has a negative effect on *mcaS* expression, most likely because it pairs with McaS, and this stimulates McaS (and *csgD* mRNA) degradation [48]. In *E. coli* C, an IS5/IS1182 transposase gene replaces the *csgD* promoter and the first ca. 30 nt of the long 5′-UTR of the gene. Albeit the transposase gene is transcribed in the same direction as *csgD* [14], the *csgD* expression profile and *csgD* mRNA abundance are in all probability different in C-1a and MG1655, and this, in turn, may affect McaS.

We found that *mcaS* is positively regulated by CsrA, as its expression decreases in both C-1a and MG1655 Δ*csrA* mutants, whereas it increases upon CsrA ectopic expression from a plasmid. This result is in contrast with previous findings showing that McaS levels were comparable between MG1655 and its isogenic *csrA*::kan mutant [7]. Such discrepancy may be due to the leakiness of the *csrA*::kan allele, which could only marginally affect *mcaS* expression with respect to the Δ*csrA* mutation, and/or to differences in the experimental conditions in which *mcaS* expression was measured.

In C-1a Δ*csrB* or Δ*csrC* mutants, the *csrA* mRNA from the P5 promoter decreases. The mechanism responsible for this drop is not straightforward, and, unfortunately, we could not directly assess whether the reduction in *csrA* mRNA caused a correspondent decrease in CsrA protein because CsrA level in the wildtype C-1a was under the detection limit of the anti-CsrA antibodies. However, if there would be a drop in the CsrA level as well, this may explain a puzzling result that we published some years ago, namely that in C-1a Δ*csrB* or Δ*csrC* mutants, the *pgaABCD* operon expression and PNAG production increased [25]. Moreover, given the indirect transcriptional activation of *csrB* and *csrC* by CsrA discussed before, low CsrA may prevent transcription also of *csrC* and *csrB* genes in Δ*csrB* and Δ*csrC* mutants, respectively. This could explain why neither gene is expressed when one of them is deleted, thus abolishing compensatory regulation.

The role of PNPase in *pgaABCD* regulation may also be connected to *csrA* expression modulation. Indeed, PNPase protects CsrB and CsrC from degradation (Figure 6). In doing so, PNPase may indirectly contribute to preserving *csrA* transcription efficiency. Consistent with this hypothesis, only RNase II, which restores CsrB and CsrC production, and not RNase R, which does not, prevent auto-aggregation [25]. Moreover, this interpretation may explain why the PNPase effect is much stronger in *E. coli* C than in K-12. In fact, C-1a contains less CsrA than MG1655, and this could make the Csr regulatory system less robust toward fluctuations of CsrA concentration.

Park and colleagues [46] showed that in vitro*,* CsrA binds the *pnp* mRNA and represses its translation and that a translational fusion encompassing the *pnp* promoter and 5′-UTR fused with the *lacZ* gene is activated in a *csrA*::kan mutant. However, in our experimental conditions (i.e., exponential cultures in M9-LG at 37 °C), neither the leaky *csrA*::kan or the Δ*csrA* mutations affect PNPase level, which is remarkably similar also between *E. coli* C and K-12 strains. Further analyses are required to assess in which conditions CsrA-dependent *pnp* regulation actually occurs in *E. coli*.

## Figures and Tables

**Table 1:** Bacteria and plasmids.

Name	Relevant Characters ^a^	Source/Reference
Bacterial Strains		
C-1a	*E. coli* C, prototrophic	[29]
C-5691	C-1a ∆*pnp751*	[30]
C-5736	∆*pgaC*	This work
C-5737	C-5691 ∆*pgaC*	This work
C-5738	C-5736 ∆*csrA*::kan	This work
C-5739	C-5737 ∆*csrA*::kan	This work
C-5741	C-1a *csrA*::kan	This work
C-5936	∆*pgaC*::kan	[25]
C-5937	C-5691 ∆*pgaC*::kan	[25]
C-5938	C-1a ∆*csrA*::kan	This work
C-5939	C-1a ∆*csrA*	This work
C-5940	∆*csrB*::kan	[25]
C-5942	C-5691 ∆*csrB*::kan	[25]
C-5944	∆*csrC*::cat	[25]
C-5946	C-5691 ∆*csrC*::cat	[25]
JW1007	BW25113 ∆*pgaC*::kan	[31]
KG-206	MG1655 ∆*pnp751*::kan	This work
KG-211	MG1655 ∆*pnp751*	This work
KG-292	MG1655 ∆*pgaC*::kan	This work
KG-293	KG-211 ∆*pgaC*::kan	This work
KG-294	MG1655∆*pgaC* ∆*csrA*::kan	This work
KG-295	KG-211 ∆*pgaC* ∆*csrA*::kan	This work
KG-299	MG1655∆*csrC*::cat	This work
KG-302	MG1655∆*csrB*::kan	This work
KG-305	KG-211 ∆*csrB*	This work
KG-307	MG1655 ∆*pgaC*	This work
KG-308	KG-211 ∆*pgaC*	This work
KG-309	KG-211 ∆*csrC*::cat	This work
MG1655	*rph* ^-^	[32]
MG1655 *csrA*::kan	*csrA*::kan	[15]
Plasmids		
pBAD21Δ1	Cloning vector	[25]
pBADpnp	pBAD24 derivative; carries the *pnp* gene	[25]
pBADrnb	pBAD24 derivative; carries the *rnb* gene	[25]
pBADrnr	pBAD24 derivative; carries the *rnr* gene	[25]
pCP20	FLP encoding plasmid	[27]
pCSRA	pGZ119HE derivative; carries 28191692818929 region of MG1655 genome under pTAC promoter	This work
pΔLpga	pJAMA8 derivative, harbors the −116 to +32 region relative to the *pgaA* transcription start site cloned into the SphI/XbaI sites	[25]
pGZ119HE	Cloning vector	[33]
pJAMA8	Cloning vector	[34]
pKD13	RED mutagenesis system plasmid	[27]
pKD3	RED mutagenesis system plasmid	[27]
pKD46	RED mutagenesis system plasmid	[27]
pLpga2	pJAMA8 derivative; carries -116 to +249 relative to transcription start of *pgaA* promoter translationally fused with *luxA*	[25]
Phage P1 hft	High transduction frequency phage P1 derivative	[35]

^a^ Coordinates refer to GenBank Accession number U00096.3.

**Table 2:** Expression of the *pgaA-lux* translational fusion.

	*E. coli* C				*E. coli* K-12			
-	wt	Δ*pnp*	Δ*csrA*	Δ*pnp* Δ*csrA*	wt	Δ*pnp*	Δ*csrA*	Δ*pnp* Δ*csrA*
LUX R.A. ^a^	1.0	14.0 ± 0.3	26.3 ± 3.0	37.9 ± 1.2	0.1 ± 0.0	0.3 ± 0.0	42.0 ± 13.5	65.1 ± 27.2
P_wt_^b^	na	9 × 10^−8^	6 × 10^−5^	4 × 10^−7^	na	1 × 10^−5^	3 × 10^−3^	7 × 10^−3^
P_C-K_^c^	3 × 10^−8^	7 × 10^−8^	ns	ns	3 × 10^−8^	7 × 10^−8^	ns	ns

^a^ Luciferase relative activity (R.A.) with respect to *E. coli* C wt strain. Results are the average of three determinations on independent cultures of the strains: *E. coli* C C-5936 (wt), C-5937 (Δ*pnp*), C-5738 (Δ*csrA*) and C-5739 (Δ*pnp* Δ*csrA*); *E. coli* K-12 KG-292 (wt), KG-293 (Δ*pnp*), KG-294 (Δ*csrA*) and KG-295 (Δ*pnp* Δ*csrA*). All strains carried Δ*pgaC* mutation and plasmid pLpga2 [25]. ^b^ *t*-test performed between determinations in each wt strain and its isogenic mutants. na, not applicable. ^c^ *t*-test performed between data relative to *E. coli* C and K-12 strains with the same mutations. ns, not significant.

**Table 3:** Expression of *csrA*, *csrB*, *csrC*, and *mcaS*.

Gene	RNA Relative Amount ^a^	
	C-5691	MG1655
*csrA* ^b^	0.3 ± 0.1	3.5 ± 1.3
*csrB*	0.2 ± 0.2	57.6 ± 9.9
*csrC*	0.1 ± 0.1	10.5 ± 5.2
*mcaS*	0.7 ± 0.1	0.2 ± 0.1

^a^ Relative amount with respect to C-1a. Cultures were grown, and Northern blotting performed as described in Figure 4A legend. Northern blot signals were quantified with ImageQuant, and the values were normalized for those of the 5S rRNA and for the C-1a values. Average of the results of three independent experiments are shown with standard deviation. For McaS, data are the average of two independent determinations with range. ^b^ The sum of signals corresponding to P3, P4, P5 mRNAs (Figure 4A) was considered for MG1655. P5 signal was considered for C-1a.

## Supplementary Materials

The following are available online at https://www.mdpi.com/article/10.3390/microorganisms9051010/s1, Table S1. Oligonucleotides. Figure S1. Growth curves of *csrA* mutants. Figure S2. Activity of the *pgaABCD* operon promoter. Figure S3. Transcription of *csrA* locus in *csrA*::kan mutants and characterization of CsrB sRNA.
