# Supplementary material for: Different csrA Expression Levels in C versus K-12 E. coli Strains Affect Biofilm Formation and Impact the Regulatory Mechanism Presided by the CsrB and CsrC Small RNAs
Source: Microorganisms. 2021 May 7;9(5):1010. doi: 10.3390/microorganisms9051010 (PMC8151843; doi:10.3390/microorganisms9051010)
Supplement: Supplementary file 1 [file microorganisms-09-01010-s001.zip › microorganisms-1210396-supplementary.pdf]

Different *csrA* Expression Levels in C versus K-12 *E. coli* Strains Affect Biofilm Formation and Impact the Regulatory Mechanism Presided by the CsrB and CsrC Small RNAs

This file contains:

Table S1. Oligonucleotides

Figure S1. Growth curves of *csrA* mutants

Figure S2. Activity of the *pgaABCD* operon promoter

Figure S3. Transcription of *csrA* locus in *csrA*::kan mutants and characterization of CsrB sRNA

Table S1. Oligonucleotides

Name	Sequence
FG2524	AGCGCCTTGTAAGACTTCGCGAAAAAGACGATTCTATCTTCGTCGACAGGTGTAGGC TGGAGCTGCTTCG
FG2525	AGGGAGCACTGTATTCACAGCGCTCCCGGTTCGTTTCGCAGCATTCCAGCATTCCGGG GATCCGTCGACC
FG2530	CGTTGTCTGACTCCCTGTCGAC
FG2531	GCTCCCTGCTCATCCTTGACAACTTT
FG2568	CCGGACGTTTGTCTTCCTG
FG2585	AAAATGTGCAAATACTGATGGCGGTTGATTGTTTGTTTAAAGCAAAGGCGTGTGTAG GCTGGAGCTGCTT
FG2586	TGCGGCGGAATCTAACAGAAAGCAAGCAAAGAAAAAAGGCGACAGATTAACATAT GAATATCCTCCTTAG
FG2624	CGGGATACAGAGAGACCCGACTCTTTTAATCTTTCAAGGAGCAAAGAATGATTCCGG GGATCCGTCGACC
FG2625	CTCACCGATAAAGATGAGACGCGGAAAGATTAGTAACTGGACTGCTGGGATGTAGG CTGGAGCTGCTTCG
FG2647	CTAATACGACTCACTATAGGGCGCCAATACGTACCTGGTTG
FG2753	ATGCGATATCCTACTTCGCGGCTACGACC
FG3151	CGAAAGCTTTAATCTTTCAAGGAGGAAAG
FG3152	CGTGGATCCCGTCTCACCGATAAAGATG
PL101	TGTCGTCAGCTCGTGTCGTGA
PL102	ATCCCCACCTTCCTCCGGT
PL191	ATGCTGATTCTGACTCGTCGAG
PL208	GGGAGTCAGACAACGAAGTG

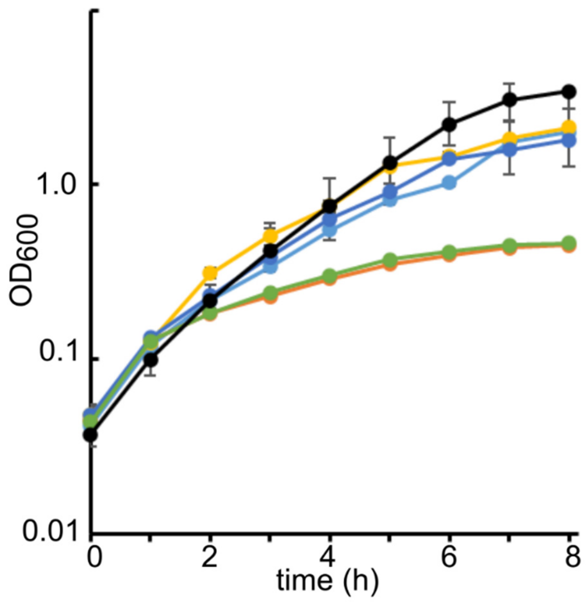

Figure S1. Growth curves of *csrA* mutants. Growth at 37 °C in M9-LG monitored by measuring the OD_{600} of cultures of the strains indicated in the Figure. *E. coli* C: *csrA*⁺, C-1a (black curve); Δ*csrA*, C-5939 (light blue curve); *csrA::kan*, C-5741, (green, orange and blue curves); *E. coli* K-12: MG1655 *csrA*::kan (yellow curve). Three C-5741 independent cultures were analysed in this experiment.

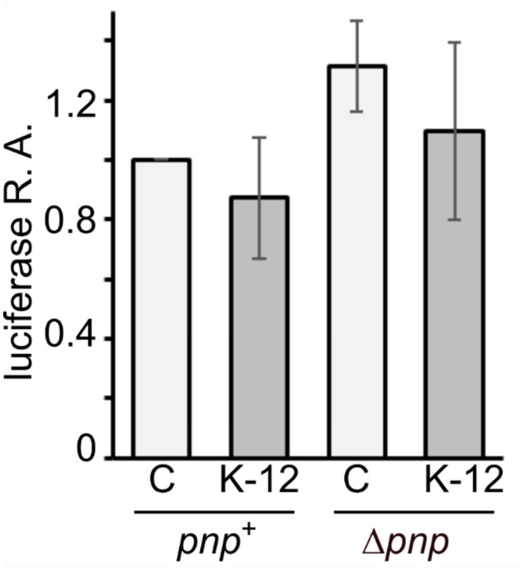

Figure S2. Activity of the *pgaABCD* operon promoter. Luciferase relative activity (R.A.) with respect to *E. coli* C wt strain. Results are the average of three determinations on independent cultures of the strains: *E. coli* C C-1a (*pnp*$^{+}$), C-5691 (Δ*pnp*); *E. coli* K-12 MG1655 (*pnp*$^{+}$), KG-211 (Δ*pnp*). All strains carried plasmid pΔLpga, which carries the pgaABCD promoter upstream of the luciferase gene [23]. Differences in luciferase R.A with respect to the C-1a are not statistically significative according to t-test.

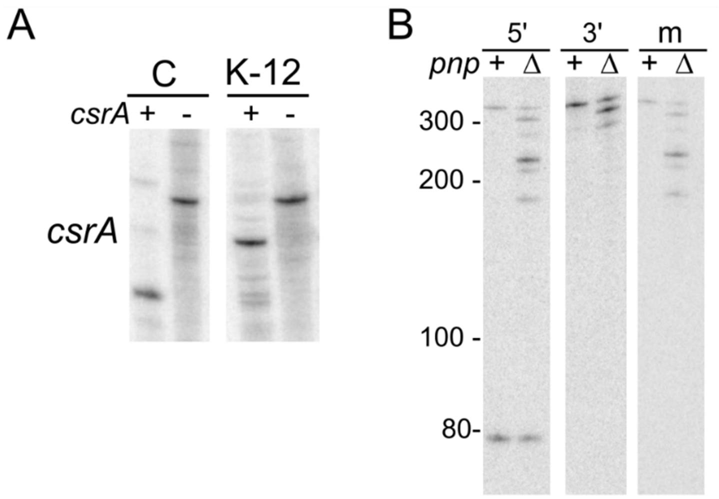

Figure S3. Transcription of *csrA* locus in *csrA*::kan mutants and characterization of CsrB sRNA. Northern blotting of RNA extracted from cultures grown in M9-LG to OD_{600} = 0.8 at 37 °C. RNA samples (10 µg) were loaded on 6% polyacrylamide-urea gel, blotted onto a nylon membrane, and hybridized with the CSRA riboprobe (**A**) or radiolabelled oligonucleotides specific for different regions of the CsrB sRNA (FG2530, CsrB 5'-end; FG2531, CsrB 3'-end; PL208, CsrB internal region, m). (**B**). **A.** RNA extracted from cultures of *E. coli* C C-1a (+, *csrA*$^+$), C-5741 (-, *csrA*::kan*)*, and *E. coli* K-12 MG1655 (+, *csrA*$^+$) and its isogenic *csrA*::kan mutant (-). **B.** RNA extracted from cultures of C-1a (+, *pnp*$^+$) and C-5691 (Δ, Δ*pnp*), KG-294 (Δ*csrA pnp*$^+$). The position and MW (in nt) of bands of the Low-range ssRNA ladder (NEB) is reported on the left.
